# Post-mortem genetic investigation of cardiac disease–associated genes in sudden infant death syndrome (SIDS) cases

**DOI:** 10.1007/s00414-020-02394-x

**Published:** 2020-08-12

**Authors:** Jasmin Köffer, Stefanie Scheiper-Welling, Marcel A. Verhoff, Thomas Bajanowski, Silke Kauferstein

**Affiliations:** 1 Institute of Legal Medicine, University of Frankfurt, Kennedyallee 104, 60596 Frankfurt/Main, Germany; 2 Institute of Legal Medicine, University of Essen, Hufelandstraße 55, 45122 Essen, Germany

**Keywords:** Sudden infant death syndrome, SIDS, Genetic heart disease, Next-generation sequencing, Targeted sequencing, Molecular autopsy

## Abstract

The sudden infant death syndrome (SIDS) is one of the leading causes of postneonatal infant death. It has been shown that there exists a complex relationship between SIDS and inherited cardiac disease. Next-generation sequencing and surveillance of cardiac channelopathy and cardiomyopathy genes represent an important tool for investigating the cause of death in SIDS cases. In the present study, targeted sequencing of 80 genes associated with genetic heart diseases in a cohort of 31 SIDS cases was performed. To determine the spectrum and prevalence of genetic heart disease associated mutations as a potential monogenic basis for SIDS, a stringent variant classification was applied and the percentage of rare (minor allele frequency ≤ 0.2%) and ultra-rare variants (minor allele frequency ≤ 0.005%) in these genes was assessed. With a minor allele frequency of ≤ 0.005%, about 20% of the SIDS cases exhibited a variant of uncertain significance (VUS), but in only 6% of these cases, gene variants proved to be “potentially informative.” The present study shows the importance of careful variant interpretation. Applying stringent criteria misinterpretations are avoided, as the results of genetic analyses may have an important impact of the family members involved.

**Electronic supplementary material:**

The online version of this article (10.1007/s00414-020-02394-x) contains supplementary material, which is available to authorized users.

## Introduction

The sudden infant death syndrome (SIDS) is still one of the leading causes of postneonatal infants death [1]. SIDS is defined as sudden death of an infant under 1 year of age, and the cause of death still remained unexplained after autopsy [2]. In most cases, death occurs during sleep. The peak incidence is between 2 and 4 months and is higher in male than in female infants [3].

A “triple risk model” for SIDS has been proposed and implies that the risk of SIDS increases if three factors concur: (1) a vulnerable infant, (2) a critical period in the development during the first months after birth, and (3) exogenous stress factors such as sleeping in prone position, smoking of the parents, and premature birth [1, 4]. Despite some successful campaigns to reduce these risk factors, SIDS is still the leading cause of death in postneonatal infants [5].

Genetic variants affecting autonomic functions, neurotransmission, energy metabolism, and cardiac repolarization have been suggested to contribute to SIDS [6–9]. These studies also showed that there exists a complex relationship between SIDS and inherited cardiac diseases. Long QT syndrome (LQTS), short QT syndrome (SQTS), Brugada syndrome (BrS), catecholaminergic polymorphic ventricular tachycardia (CPVT), and hypertrophic cardiomyopathy (HCM) have been described as a monogenic cause in a certain number of SIDS cases [6, 9–14].

The development of next-generation sequencing (NGS) offers new opportunities to investigate potential pathogenic sequence variations as an underlying cause of death in SIDS cohorts. Several studies suggest that in up to 30% of SIDS cohorts pathogenic mutations in cardiac channelopathy–associated genes contributed as a monogenic cause of death [4, 9, 12, 13].

In 3.5% of SIDS cases, a pathogenic variation in a cardiomyopathy-associated gene was detected [14].

The detection of variants and its evaluation is important when counseling the family members in a SIDS case, but special precautions have to be made when a variant is considered to be pathogenic. In a recent study of variant analysis, 4.3% of pathogenic variations and 13% of “informative variations” have been found, which is a much lower percentage than that reported in previous studies [2].

In the present study, an analysis of genes associated with genetic heart diseases in a cohort of 31 SIDS cases was performed to investigate the presence of potentially causative variants, which may represent a predisposing risk factor for sudden death in infancy.

## Methods

### Study group

The SIDS cohort consisted of 31 SIDS cases (Table 1). Pulmonary tissue samples had been collected during autopsy between 1999 and 2001 as part of a study on SIDS (GeSID) by Findeisen et al. [15]. These samples were stored at − 20 °C. The following criteria applied: (1) sudden unexplained death of an infant < 1 year of age, (2) no conclusive results of autopsy, (3) negative toxicology analysis, and (4) no pathological microscopic findings, except signs of cardiomyopathy. The study was approved by the local Ethic committee (protocol number E84/06).

**Table 1:** Summary of the sudden infant death syndrome cohort

Case	Sex	Age
1	m	7 weeks
2	f	7 weeks
3	m	2.5 months
4	m	3 months
5	f	3 months
6	m	1.5 months
7	f	2 months
8	f	4.5 months
9	f	5 months
10	m	6 months
11	f	2 months
12	m	3 weeks
13	m	9 months
14	m	4 months
15	f	6 weeks
16	m	6.5 months
17	m	4 months
18	m	3 months
19	f	2.5 months
20	f	3 months
21	m	13 days
22	f	2 months
23	f	5 weeks
24	f	9 months
25	m	3 months
26	m	2 months
27	m	4 months
28	m	2 months
29	f	2.5 months
30	m	7.5 months
31	m	4 weeks

### Targeted sequencing

Genomic DNA was isolated from stored tissue samples using phenol-chloroform extraction. The quality and quantity of DNA was assayed using the Nanodrop® ND-1000Spectromphotometer v3.1.0 (Thermo Fisher Scientific) and the Qubit Fluorometer 3.0 with dsDNA BR and HS assay kits (Invitrogen, Thermo Fisher Scientific), respectively. DNA integrity was assessed using the genomic DNA ScreenTape and Agilent 4200 TapeStation system.

Paired-end libraries were prepared following the manufacturer’s protocol Nextera™ Flex for Enrichment (Illumina), and the TruSight cardio panel (Illumina) consisting of 174 genes with known cardiac associations was applied. Since the samples had been stored for a long period of time at − 20 °C and some samples showed DNA degradation, the recommendations for formalin-fixed paraffin-embedded (FFPE) samples in the protocol were followed. For samples exhibiting a DNA integrity number (DIN) lower than 5, 300 ng of DNA was used as input for tagmentation, above a DIN of 5, 200 ng was used.

In order to obtain a better performance in GC-rich regions, the PCR program “amplify tagmented DNA” was modified and denaturation times in steps 2 and 3 were prolonged (4 min and 30 s instead of 3 min and 30 s, respectively). Hybridization of probes was performed overnight. Enriched libraries were amplified with adjusted doubled denaturation times in steps 1 and 2 of the protocol. The genomic DNA sample NA12878 (Coriell Institute) was used as a control in each sequencing run.

Sequencing was performed on the Illumina® MiniSeq™ system.

### Variant analysis

Resulting reads were aligned to the GRChr37 (hg19) human reference genome. GensearchNGS software (Phenosystems ®) was used for evaluation of the data. The genetic data were filtered for aberrations in genes associated with cardiac channelopathies and cardiomyopathies (*n* = 80, supplemental data) and according to a pre-established prioritization protocol mainly based on presumed functional impact on the protein and allele frequency. The resulting data were evaluated bioinformatically, and detected sequence variants were assessed using common databases (The Genome Aggregation Database, NHLBI Exome Sequencing Project, NCBI dbSNP, Human Gene Mutation Database) and applying in silico prediction tools (PolyPhen-2 [16], MutationTaster [17] , SIFT [18], and CADD [19]).

Furthermore, two different minor allele frequencies (MAFs) were applied and the results compared. The MAF is the second most frequent allele value and is used to distinguish common polymorphisms from rare variants. The MAF of 0.2% reflects the prevalence of hypertrophic cardiomyopathy (HCM), known to be responsible for some SIDS cases. The MAF of 0.005% (1:20000 alleles) was used to evaluate “ultra-rare” variants based on a recent genetic SIDS-study [2]. For this evaluation, the minor allele frequency data of the Genome Aggregation Database (gnomAD) were utilized.

Filtered rare variants were classified according to the standards of the American College of Medical Genetics and Genomics (ACMG) [20]. Genetic variants expected to disrupt protein function (nonsense, frameshift, splice site variants, missense variants with established functional evidence) or rare missense variants within the core genes (supplemental data) were considered to be important variants (VUS, potentially informative), when they were located in a functional important domain, a mutation hotspot or described in this context in the literature.

## Results

### SIDS cohort

**Fig. 1:**
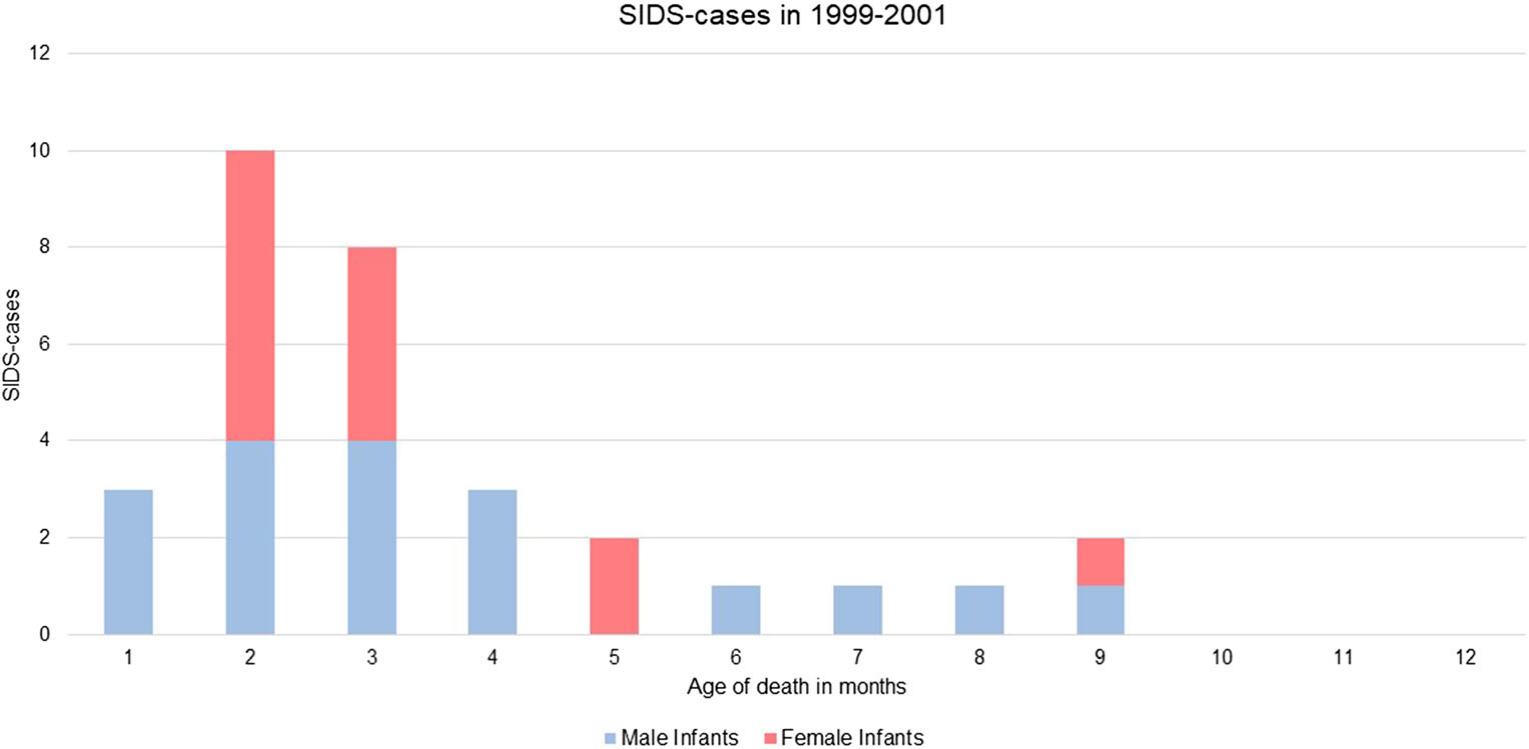
Occurrence of sudden infant’s death within the first year of life

Targeted sequencing was performed in 31 SIDS cases (18 male, 13 females; average age 3.4 months), which were collected as a part of the published German study on SIDS (GeSID) in the years from 1999 to 2001. The present study showed a higher risk of SIDS at the age of 2 to 3 months (58%). There were no cases of SIDS at the age of 10 to 12 month (Fig. 1). The data represent a higher risk of SIDS in males (58%), but gender distribution is equal at the age of 2–3 months.

### Sequencing results

Next-generation sequencing was successfully performed for all 31 SIDS cases with an average depth of coverage of 490x. For stringent variant classification, a standardized protocol was used. Filtering with a MAF of  ≤ 0.2%, a total of 14 rare variants were identified. After filtering with a MAF of ≤ 0.005%, only 8 ultra-rare sequence variations were found. The potential significance of all sequence variations was assessed and classified according to the ACMG standards. Variants classified as uncertain significance were further divided in subclasses (VUS, probably benign and VUS, potentially informative) to better evaluate their potential relevance.

**Fig. 2:**
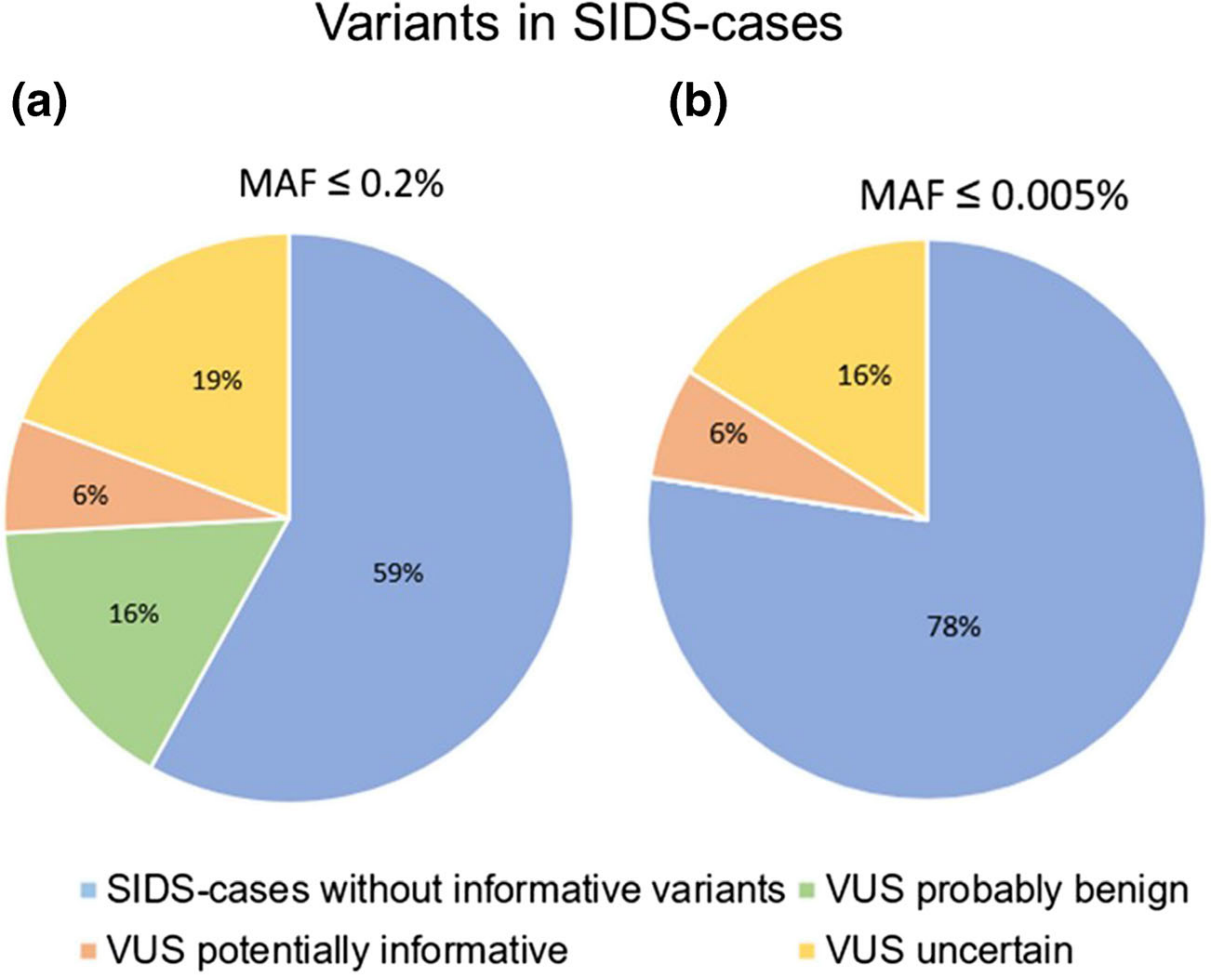
Percentage of variants associated with genetic heart diseases in SIDS cases. **A** With a minor allele frequency (MAF) ≤ 0.2%. **B** With a minor allele frequency (MAF) ≤ 0.005%

Rare variants were detected in 9 and ultra-rare variants in 8 of the 80 genes, respectively (Table 2). The distribution of these subclasses between the different MAFs is shown in Fig. 2.

**Table 2:** Summary of variants identified in sudden infant death syndrome cases

SIDS case	Age	Sex	Gene	Nucleotide change	Amino acid change	CADD	Polyphen	SIFT	MutationTaster	MAF (%)	ACMG
#1	7 weeks	m	SCN5A	c.3947 G > A (NM_198056)	p.(Arg1316GIn)	24.8	PoD	D	Disease causing	0.003	VUS
#2	2.5 months	m	RBM20	c.3595 G > A (NM_001134363)	p.(G1u1199Lys)	23.4	B	D	Disease causing	0.003	VUS
#6	1.5 months	m	CSRP3	c.149 C > A (NM_003476)	p.(Ala50Glu)	24.3	PrD	D	Disease causing	0.0007	VUS
#7	2 months	f	MYH6	c.4505 G > A (NM_002471)	p.(Arg1502GIn)	32	PrD	D	Disease causing	0.016	VUS
#15	6 weeks	f	JUP	c.1130 G > A (NM_002230)	p.(Arg377His)	29.8	PrD	D	Disease causing	0.005	VUS
#19	2.5 months	f	MYH7	c.4075 C > T (NM_000257)	p.(Arg1359Cys)	29.5	PrD	D	Disease causing	0.0008	VUS
			KCNJ2	c.47 A > G (NM_000891)	p.(Glu16Gly)	28.7	PoD	D	Disease causing	0.0004	VUS
#22	2 months	f	PRKAG2	c.1508 A > G (NM_016203)	p.(GIn503Arg)	25.5	PoD	T	Disease causing	0.001	VUS
#21	4 months	m	LMNA	c.632 A > G (NM_170707)	p.(Tyr211Cys)	24.6	PoD	T	Disease causing	0.003	VUS

By applying a MAF of 0.2%, 13 (41%) cases with 14 rare variants were identified, with a MAF of 0.005%, only 7 (22%) cases with 8 ultra-rare variants were obtained. For variants considered to be “potentially informative” (6%), no differences between the two (MAFs) allele frequency filters were observed. Moreover, with MAF of 0.005%, in 16% variants were classified in a non-informative context; with MAF of ≤ 0.2%, these variants were classified to exhibit uncertain significance (19% instead of 16%) or as benign (16%).

### Genetic findings

In case 1, one potentially informative variant p.(Arg1316Gln) was identified in the *SCN5A*-gene. In this case, no abnormalities of the heart have been found at autopsy and no cardiac disease history was reported by the family. The twin brother of the deceased infant exhibited no pathological cardiac symptoms. Abnormalities in the gene *SCN5A* are mainly associated with BrS and LQTS [21]. The detected variant is located at the transition site of the sodium channel in the fourth segment of the third domain close to the cytoplasm and has not been described in the literature and functional studies so far. In the database ClinVar the sequence variant was reported once in conjunction with BrS and was categorized as VUS. However, this variant may have functionally important, deleterious effects. The CADD-Score of 24.8 indicates that the nucleotide change may be of functional relevance. However, according to the ACMG guidelines, the potentially informative variant has to be classified as variant of uncertain significance due to missing functional characterization studies as well as co-segregation data.

In case 19 no pathological abnormalities of the heart had been detected at autopsy. According to the family history, another SIDS case had occurred in the year before. One variant, p.(Arg1359Cys), was detected in the *MYH7*-gene, which has been described to be associated with cardiomyopathy. In ClinVar the variant is categorized as variant of uncertain significance. The high CADD-Score (29.5) suggests a pathogenic effect. The variant was classified as potentially informative VUS based on the criteria as shown above. According to Hershberger et al. [22] and Klaassen et al. [23], this variant has been found in patients with cardiomyopathy and is considered to be potentially pathogenic.

The second variant in this case p.(Glu16Gly) was detected in the *KCNJ2*-gene. This gene is associated with LQTS, SQTS, and CPVT [24, 25], and the detected variant has not been described in ClinVar so far. The high CADD-Score (28.7) suggests pathogenicity. Based on the ACMG guidelines, the variant was classified as VUS, since the variant is not localized in a functional important domain and no co-segregation data are reported or functional studies exist.

Following the strict ACMG guidelines and a conservative MAF, 2 of the ultra-rare variants present in two (6%) of the 31 SIDS cases were classified as VUS with potentially pathogenic impact.

## Discussion

Although the incidence of the sudden infant death syndrome (SIDS) drastically decreased during the last years, it is still one of the leading causes of death in industrialized countries [5]. SIDS is a multifactorial and heterogeneous incident, which may be associated with variants in various genes.

Inherited channelopathies as well as cardiomyopathies have been considered as a monogenic cause in some SIDS cases [2]. In studies of the genetic background of SIDS victims next-generation sequencing technologies represent a novel approach [26–28].

In the present study, postmortem genetic screening of 80 candidate genes was performed in selected SIDS cases in order to detect the presence of potentially causative variants in a spectrum of cardiac arrhythmia syndrome–related genes. The study included a cohort of 31 SIDS infants with a peak incidence between 2 and 4 months of age and slightly higher prevalence in boys (58%), which is in line with previous studies [2, 3, 5].

Although the study confirms the high potential of NGS to identify informative variants, a careful and stringent variant analysis is essential to enable appropriate counseling of the families involved.

Therefore, evaluating the sequencing data using a threshold for allele frequency is one of the most important criteria in an accurate data analysis. Since prevalence of SIDS is 1:5000 in live births and causative disorders are rare, two different MAF cutoffs were used in the present study: 0.2%, which is based on the prevalence of hypertrophic cardiomyopathy (1:500) and 0.005% (1 in 20,000 alleles or 1 in 10,000 individuals), reflecting rare variants as recommended in a study by Tester et al. [2]. By applying these two allele frequency filters, we tried to minimize the problem of incorrect over-interpretation of presumably pathogenic variants and the possibility of excluding potentially pathogenic variants that are rare. Using this strategy, 25% of cohort members possessed a rare genetic variant. In two cases (6%), the variants were classified as potentially pathogenic variants of uncertain significance. However, despite their rarity, the pathogenic significance of the variants is still unknown. Neubauer et al. [4] detected variants with potentially pathogenic effects in 20%, Hertz et al. [29] in 34% of SIDS cases, respectively. In both studies, a relatively high allele frequency of < 1% for filtering of variants was used. However, these rather high values in the assessment of pathogenic variants are probably overestimating their relevance in respect of the rarity SIDS represents. The data of the present study rather support those obtained by Tester et al. [2], although the frequency of informative variants is significantly lower. The lower allele frequency of 0.005% leads to potentially informative variants in only 2 (6%) out of 31 cases. These differences clearly show the need of a standardized practice for the interpretation of rare variants associated with inherited diseases.

### Allele frequency

The comparison of probably benign variants indicates that they are omitted by lower allele frequency and therefore are not rare. Based on previous results, filtering with a low allele frequency is useful for assessing variants, but one has to keep in mind that potentially important variants and information with regard to genetic modifiers may be missed, which could play a role in SIDS, but are too common to be detected with the filter applied.

### Study limitations

Based on the size of the gene Titin (*TTN*) and the high number of missense variants with limited clinical value, detected missense *TTN* variants were not further assessed.

Since the samples had been collected anonymously, the families related with the cases exhibiting probably informative variants could not be informed. Therefore, important results of cardiological and genetic investigations of family members for co-segregation studies were not possible.

## Conclusion

Targeted sequencing using a cardiac gene-specific focus reveals that about 20% of 31 SIDS cases exhibited a variant of uncertain significance. By applying a stringent variant classification, only 6% of these cases showed a potentially pathogenic variant. The study clearly points to the importance of careful variant interpretation. Results of genetic analyses may have an important impact on the family members involved.

## Electronic supplementary material

ESM 1 (XLSX 11.7 kb)
